# Melanocortin-1 receptor expression as a predictive factor for postoperative outcomes in melanoma patients: a retrospective study

**DOI:** 10.3389/fimmu.2025.1570502

**Published:** 2025-03-27

**Authors:** Tian Xiang, Haiying Li, Xiaowei Wang, Danke Su

**Affiliations:** ^1^ Department of Radiology, Guangxi Medical University Cancer Hospital, Guangxi Medical University, Nanning, Guangxi, China; ^2^ Department of Nuclear Medicine, The Second Xiangya Hospital, Central South University, Changsha, Hunan, China; ^3^ Department of Pathology, The Second Xiangya Hospital, Central South University, Changsha, Hunan, China

**Keywords:** melanoma, MC1R, tumor site, immunotherapy, prognosis

## Abstract

**Background and objective:**

This study aims to explore the relationship between melanocortin-1 receptor (MC1R) expression levels and clinical pathological parameters of melanoma, as well as its potential as a prognostic biomarker.

**Methods:**

This retrospective study included 99 melanoma patients in our hospital from June 2017 to July 2023. MC1R expression was assessed by immunohistochemistry assays. Histochemistry score (H-score) determined the level of MC1R immunohistochemistry expression in melanoma. The relationships among MC1R expression, clinical pathological parameters in melanoma patients were assessed using Chi-square and Fisher’s precision probability tests. Kaplan-Meier assay and log-rank test were utilized to estimate survival curves. Potential independent factors among the enrolled patients were investigated using COX regression analysis.

**Results:**

According to median value of H-score, 38 cases with low MC1R expression and 61 cases with high MC1R expression in melanoma tumor tissues were observed. Patients with high MC1R expression in melanoma tissues exhibited a worse prognosis compared to patients with low MC1R expression. The survival time difference was statistically significant [MC1R expression in melanoma tumor tissue (MC1RT): median DFS, 12.83 vs. 17.53 months, χ2 = 5.395, P=0.0202; median OS, 16.47 vs. 21.77 months, χ2 = 5.082, P=0.0243. MC1R expression in normal adjacent to melanoma tissue (MC1RN): median DFS, 12.03 vs. 14.29 months, χ2 = 6.864, P=0.0088; median OS, 16.73 vs. 21.77 months, χ2 = 5.649, P=0.0175]. Multivariate COX regression model analysis indicated that MC1RN, MC1RT, sex, ESR, tumor site, targeted therapy, and immunotherapy were potential prognostic factors for the DFS. Furthermore, MC1RN, MC1RT, sex, tumor site, TLN, PLN, and immunotherapy were potential prognostic factors for the OS. Calibration curve indicated the predicted probabilities of nomogram models were in accordance with the actual probabilities, and the prediction accuracy was relatively high at one year and three years following surgery. The decision clinical curve revealed that the nomogram models had better predictive performance for DFS and OS than the MC1RT or MC1RN thresholds.

**Conclusions:**

Low MC1R expression in melanoma tumor tissues and adjacent normal tissue might be beneficial for the prognosis of melanoma patients. MC1R was a predictive factor for the prognosis of melanoma patients. Nomogram models based on MC1R demonstrated good prediction ability.

## Introduction

Melanoma is a common malignant tumor of skin, characterized by early metastasis and poor prognosis (1). Globally, more than 280,000 (1.6%) new cases of melanoma occur each year, with over 60,000 (0.6%) deaths due to this disease (2). Recently, the incidence rate of melanoma is increasing rapidly in China (3). Approximately one new case of melanoma occurs in every 300,000 individuals, and one out of every 500,000 deaths is due to melanoma (4). The primary treatment for melanoma is surgery that complemented by adjuvant therapy, such as chemotherapy or radiotherapy (5). The five-year survival rate for localized melanoma is 98%, with a 64% survival rate for regional metastasis, and distant metastasis exhibits a 23% survival rate (6).

Melanocortin-1 receptor (MC1R) was first cloned by the Chhajlani and Mountjoy research groups in 1992, and composed of 317 amino acids (5). MC1R is expressed in human melanoma cells, and plays a critical role in skin tone and hair color formation (7). Some studies have shown that MC1R expression is associated with the prognosis of tumors. For example, elevated expression of MC1R in colorectal cancer (CRC) is associated with microsatellite instability (MSI) (8). In Chelakkot’s study, cell proliferation was significantly reduced *in vitro* and *in vivo* when the MC1R gene was knocked out (9). MC1R expression could be an auxiliary molecular marker in diagnosing esophageal squamous cell carcinoma (10). Another study also indicated that elevated MC1R expression levels were significantly associated with a lower recurrence of the free-survival rate in ovarian cancer (11).

Recently, MC1R has attracted the interest of numerous researchers as a target for melanoma. Chen’s study indicated that the APT2 inhibitor ML349 increased MC1R signaling and inhibited UVB-induced melanoma formation (12). Another study shown red-hair variant of MC1R exhibited differences in the pigmentation phenotype between males and females, and patients with the red-hair MC1R variant had an increased risk of developing melanoma (13). Yang’s study demonstrated that the peptide 68Ga-DOTA-GGNe CycMSHhex could specifically bind to MC1R, and the labeled peptide revealed melanoma metastases. Moreover, this peptide could bind to MC1R on the cell surface of melanoma brain metastases, indicating that this peptide exhibited excellent potential as a signal in imaging examinations of melanoma patients with brain metastases (14). However, few studies have systematically examined MC1R expression in melanomas in patients with gender, TNM stage, or tumor sites. Thus, the ability of MC1R expression to predict the survival rate for melanoma patients is still controversial.

The current study retrospectively analyzed the expression level of MC1R in melanoma patients, and evaluated the correlation between MC1R expression and clinical pathological indicators. This study also explored whether MC1R expression could serve as a prognostic biomarker for predicting prognosis for melanoma patients.

## Materials and methods

### Patients

Ninety-nine melanoma patients admitted to our hospital from June 2017 to July 2023 were enrolled into this retrospective analysis. The enrolled patients received surgical treatment and histological specimens were obtained. This research received approval from the ethical committee of Second Xiangya Hospital of Central South University, written informed consent was obtained from each participant.

### Inclusion and exclusion criteria

Inclusion criteria included: (1) Histopathological diagnosis of melanoma; (2) Received surgical treatment and histopathological specimens were obtained; (3) A complete hematology examination, pathology, follow-up information were obtained. Exclusion criteria included: (1) Underwent anti-tumor treatment before surgery, such as chemotherapy, radiotherapy, immunotherapy; (2) Additional diseases were present, such as hypertension, diabetes, and coronary heart disease that were difficult to control; (3) Incomplete information was provided.

### Tissue processing and immunohistochemistry

Melanoma tissue samples were obtained from our hospital, and fixed in formalin and embedded in paraffin, following routine protocols. Immunohistochemistry was performed based on standard protocols as follows. (1) Paraffin removal and clearing. (2) Antigen retrieval. (3) Blocking endogenous peroxidases. (4) Serum blocking. (5) Primary antibody. MC1R antibody (ab236734, 1:200, Waltham, MA, USA) was added to the tissue sections overnight. (6) Secondary antibody. HRP-conjugated Goat Anti-Mouse IgG (H+L) (GB23301, 1:500, Wuhan, China) in a humid chamber for two hours. (7) DAB staining. The sections were rinsed, and a freshly prepared DAB colorimetric solution was added. (8) Nuclear staining. The cell nuclei in the tissue sections were stained with hematoxylin for approximately three minutes. (9) Dehydration and cover-slipping. (10) Microscopic examination. Images of the stained, cover-slipped tissue sections were assessed using Aipathwell image acquisition and analyzed.

### Immunohistochemical scoring criteria for MC1R expression

Aipathwell is a digital pathological image analysis software program based on artificial intelligence learning. Histochemistry scores (H-scores) were obtained using a histological scoring method for the immunohistochemically stained tissue sections. It converted the ratio of positively stained cells and staining intensity into corresponding values to achieve a comprehensive semi-quantitative analysis of the degree and quantity of positive tissue immunostaining. The H-Score was calculated as follows: (∑(pi×i) = weak intensity cells (%) ×1 + moderate intensity cells (%) ×2 + strong intensity cells (%) ×3. According to the scheme, **i** represented the staining intensity of the positive cells: 1) Negative = no staining, 0 point; 2) Weak positive (light yellow) = 1 point; 3) Moderately positive (brownish yellow) = 2 points; 4) Strong positive (brown) = 3 points. Pi represented the percentage of positive cells at the corresponding level. The value of H-score ranged from 0 to 300.

In the current study, we analyzed the expression level of MC1R in tumor tissues and adjacent normal tissue, respectively. These patients were divided into two groups based on the median value of H-Score. The expression level of MC1R in tumor tissues with an H-Score ≤ 6.90 indicated low expression of MC1R (38 cases), while an H-Score>6.90 indicated high MC1R expression (61 cases). Considering the MC1R expression in adjacent normal tissues, an H-Score ≤ 3.61 indicated low expression of MC1R (58 cases), and an H-Score>3.61 indicated high MC1R expression (41 cases).

### Follow-up

All enrolled patients in the study were followed through telephone interviews and outpatient visits. Follow-up assessments included routine blood analysis, blood biochemistry, coagulation function, tumor markers, magnetic resonance imaging, and others. The patients were assessed every three months after surgery. In this study, disease free survival (DFS) was referred to the duration between the time from the diagnosis of melanoma at admission and the discovery of local or distant melanoma metastasis, or the date of the last visit. Overall survival (OS) was referred to the duration between the time from the diagnosis of melanoma at admission and death from any cause or the last visit.

### Statistical methods

All statistical analyses were conducted using SPSS Statistics software 29.0 (IBM Corp, https://www.ibm.com/sps), as well as R software (version 4.2.2; Vienna, Austria URL: http://www.R-project.org/). The chi-square and Fisher’s precision probability tests were applied to assess the relationship between MC1R and the clinical pathological characteristics of melanoma patients. The Cox proportional hazards regression model was employed to identify the underlying independent variables that were associated with DFS and OS. Kaplan-Meier method was utilized for computing the survival curves of DFS and OS, followed by a comparison using the log-rank test. In view of the multivariate COX regression analysis of the potential prognostic factors, nomograms were constructed to predict the DFS and OS in the melanoma patients. Calibration curves and decision curve analyses were used to assess the calibration and practicality of the model. P<0.05 was considered statistically significant.

## Results

### MC1R expression in melanoma patients and its relationship with prognosis

**Figure 1:**
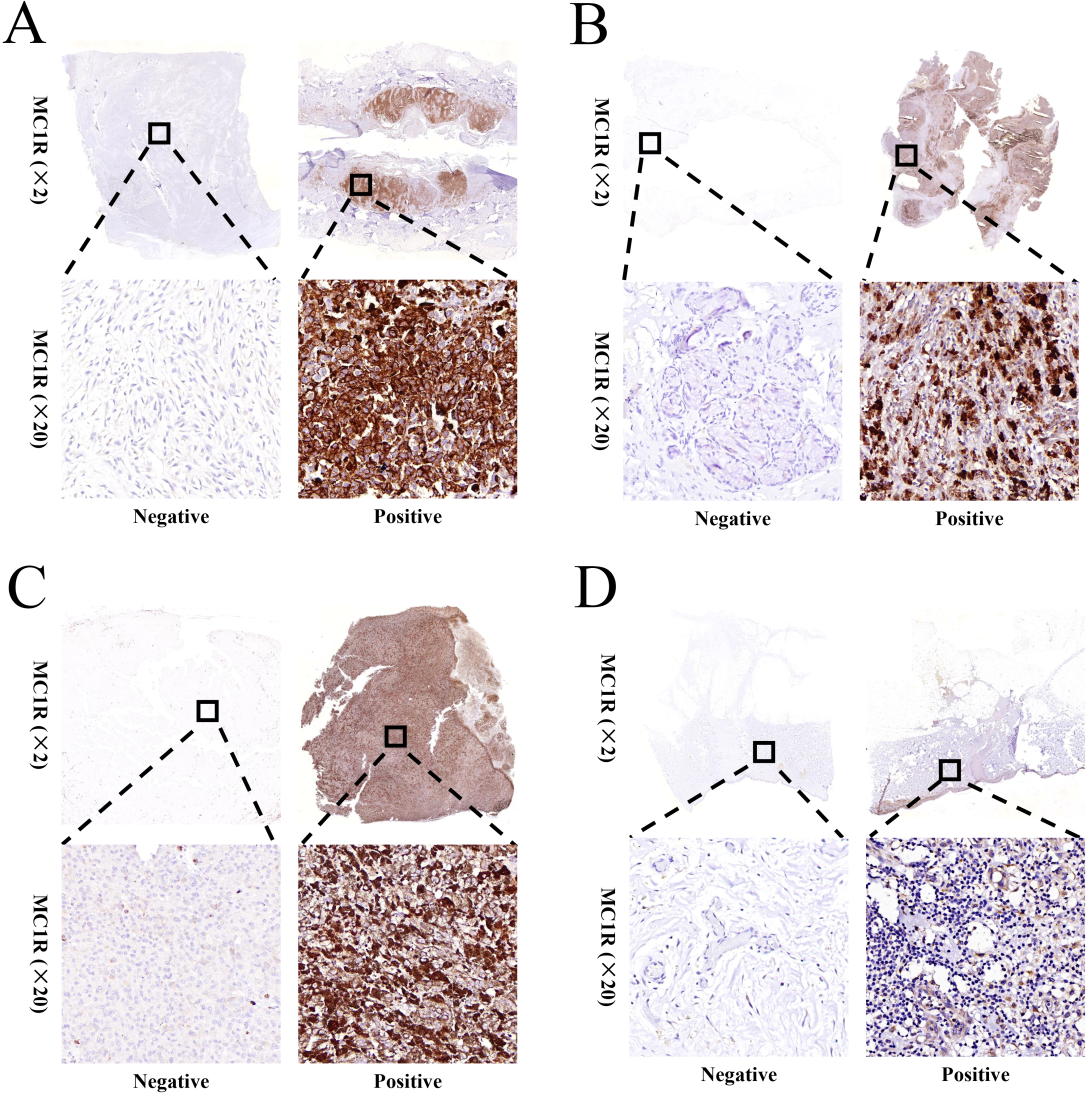
Representative images for the differential expression of MC1R in **(A)** plantar melanoma tumor tissue, **(B)** nasal melanoma tumor tissue, **(C)** ocular melanoma tumor tissue, and **(D)** dorsal surface melanoma tumor tissue.

MC1R expression was detected using immunohistochemistry for the 99 melanoma patients enrolled into this study. The expression of MC1R in melanoma tumor tissues was significantly higher than that in adjacent normal tissue, and differential expression was observed in different melanoma patients (**Supplementary Figure S1**). The representative MC1R immunohistochemistry images of melanoma patients were performed in **Figures 1A–D**.

**Figure 2:**
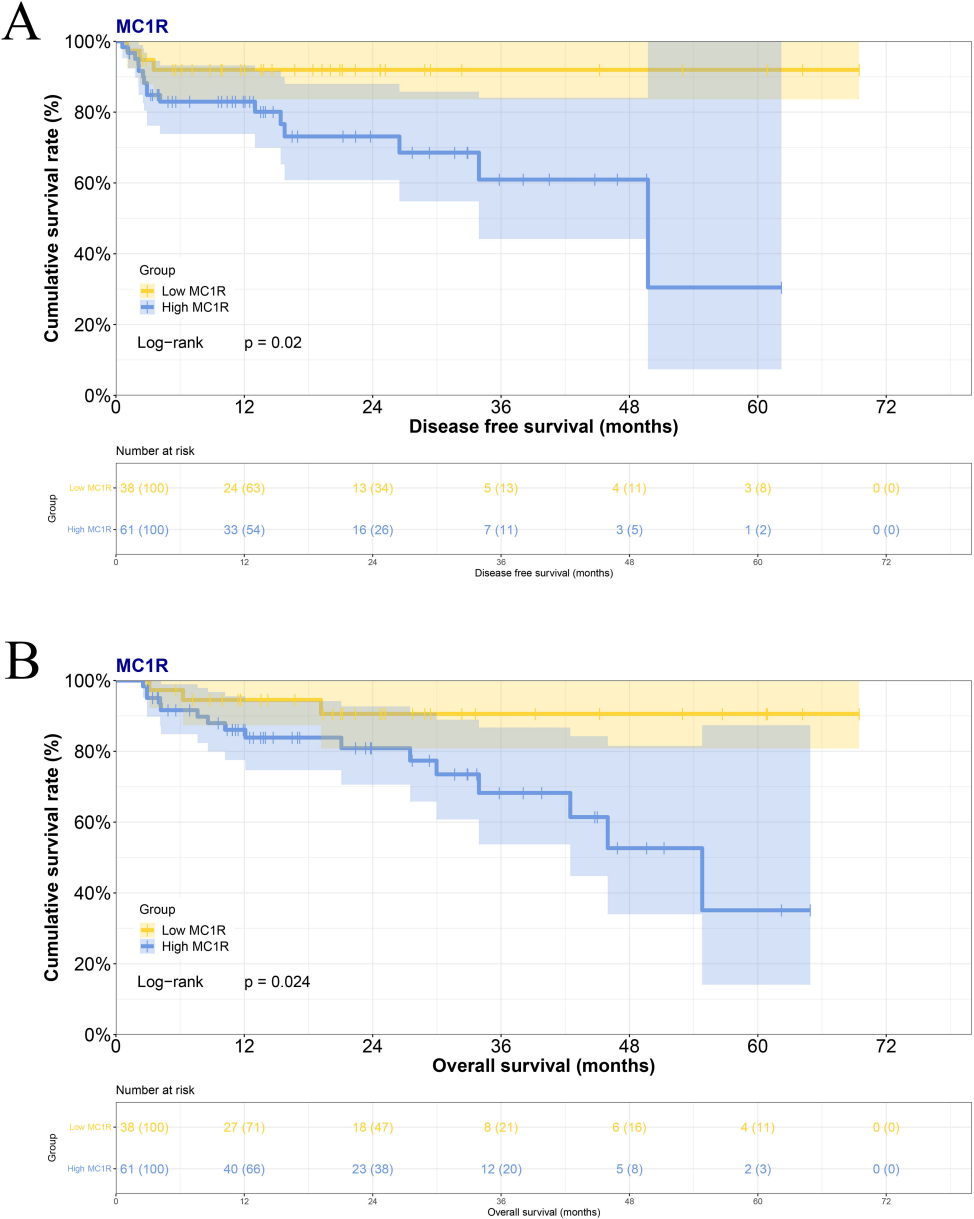
The association between MC1R expression in melanoma tumor tissues and the prognosis in melanoma patients for **(A)** DFS and **(B)** OS.

Based on the immunohistochemical staining results by H-Score, there were 38 cases with low MC1R expression and 61 cases with high MC1R expression in melanoma tumor tissues. Melanoma patients exhibiting high MC1R expression experienced a shorter survival time. The difference in survival times was statistically significant (median DFS: 12.83 months vs. 17.53 months, χ^2^ = 5.395, P=0.0202; median OS: 16.47 months vs. 21.77months, χ^2^ = 5.082, P=0.0243) (**Figures 2A, B**).

When normal tissues adjacent to melanoma sites were examined, 58 cases with low expression of MC1R and 41 cases with high MC1R expression were observed. Patients with high expression of MC1R also exhibited a shorter survival time, and the difference in survival times was statistically significant (median DFS: 12.03 months vs. 14.29 months, χ^2^ = 6.864, P=0.0088; median OS: 16.73 months vs. 21.77 months, χ^2^ = 5.649, P=0.0175) (**Supplementary Figures S2A, B**).

### Comparison of clinical and pathological characteristics in melanoma patients

The median age was 58 years, with an average age of 57.9 ± 13.2 years. There were 63 male patients and 36 female patients. Of all enrolled patients, 7 cases with stage I, 71 cases with stage II, 19 cases with stage III, 2 cases with stage IV, respectively. The distribution of tumor sites for melanoma was as follows: 21 cases (21.2%) of ocular choroid, 23 mucosal cases (23.2%), and 55 cases (55.6%) of acral skin. Every patient received surgical treatment. Furthermore, 16 cases (16.2%) received chemotherapy after surgery, eight cases (8.1%) received radiotherapy, 15 cases (15.2%) received targeted therapy, and 31 cases (31.3%) received immunotherapy. According to MC1R expression in melanoma tumor tissues, the expression of MC1R in melanoma tumor tissues was correlated with several factors, including family history, A/G, DBIL, and tumor site (**Table 1**).

**Table 1:** Clinicopathological characteristics of melanoma patients associated with the expression of MC1R in melanoma tumor tissues.

Parameters	level	Low MC1R	High MC1R	p
n		38	61	
Sex	Male	24 (63.2)	39 (63.9)	1.000
	Female	14 (36.8)	22 (36.1)	
Age	≤58	19 (50.0)	34 (55.7)	0.727
	>58	19 (50.0)	27 (44.3)	
Weight	≤60	19 (50.0)	31 (50.8)	1.000
	>60	19 (50.0)	30 (49.2)	
Height	≤1.60	17 (44.7)	28 (45.9)	1.000
	>1.60	21 (55.3)	33 (54.1)	
BMI	≤23.44	18 (47.4)	35 (57.4)	0.445
	>23.44	20 (52.6)	26 (42.6)	
Family history	Yes	6 (15.8)	1 (1.6)	0.023
	No	32 (84.2)	60 (98.4)	
ALT	≤17.65	16 (42.1)	32 (52.5)	0.426
	>17.65	22 (57.9)	29 (47.5)	
AST	≤19.50	21 (55.3)	32 (52.5)	0.948
	>19.50	17 (44.7)	29 (47.5)	
AST/ALT	≤1.10	22 (57.9)	30 (49.2)	0.524
	>1.10	16 (42.1)	31 (50.8)	
TP	≤66.55	20 (52.6)	33 (54.1)	1.000
	>66.55	18 (47.4)	28 (45.9)	
ALB	≤39.80	18 (47.4)	33 (54.1)	0.656
	>39.80	20 (52.6)	28 (45.9)	
GLO	≤26.65	26 (68.4)	30 (49.2)	0.095
	>26.65	12 (31.6)	31 (50.8)	
A/G	≤1.48	12 (31.6)	35 (57.4)	0.022
	>1.48	26 (68.4)	26 (42.6)	
TBIL	≤10.50	15 (39.5)	34 (55.7)	0.172
	>10.50	23 (60.5)	27 (44.3)	
DBIL	≤3.30	13 (34.2)	38 (62.3)	0.012
	>3.30	25 (65.8)	23 (37.7)	
TBA	≤4.55	23 (60.5)	33 (54.1)	0.675
	>4.55	15 (39.5)	28 (45.9)	
BUN	≤5.51	24 (63.2)	32 (52.5)	0.403
	>5.51	14 (36.8)	29 (47.5)	
CREA	≤67.25	18 (47.4)	34 (55.7)	0.546
	>67.25	20 (52.6)	27 (44.3)	
UA	≤325.90	18 (47.4)	36 (59.0)	0.355
	>325.90	20 (52.6)	25 (41.0)	
CO2CP	≤24.95	19 (50.0)	35 (57.4)	0.610
	>24.95	19 (50.0)	26 (42.6)	
GLU	≤4.94	19 (50.0)	34 (55.7)	0.727
	>4.94	19 (50.0)	27 (44.3)	
FDP	≤1.78	25 (65.8)	34 (55.7)	0.435
	>1.78	13 (34.2)	27 (44.3)	
INR	≤0.95	22 (57.9)	36 (59.0)	1.000
	>0.95	16 (42.1)	25 (41.0)	
Fib	≤2.88	21 (55.3)	27 (44.3)	0.391
	>2.88	17 (44.7)	34 (55.7)	
D Dimer	≤0.38	24 (63.2)	35 (57.4)	0.719
	>0.38	14 (36.8)	26 (42.6)	
AT III	≤94.40	17 (44.7)	30 (49.2)	0.823
	>94.40	21 (55.3)	31 (50.8)	
HbA1c	≤6.50	26 (68.4)	34 (55.7)	0.296
	>6.50	12 (31.6)	27 (44.3)	
ESR	≤8.00	27 (71.1)	35 (57.4)	0.248
	>8.00	11 (28.9)	26 (42.6)	
CRP	≤3.59	19 (50.0)	31 (50.8)	1.000
	>3.59	19 (50.0)	30 (49.2)	
Blood type	A	14 (36.8)	16 (26.2)	0.163
	B	10 (26.3)	24 (39.3)	
	O	14 (36.8)	17 (27.9)	
	AB	0 (0.0)	4 (6.6)	
White blood cell	≤6.10	22 (57.9)	27 (44.3)	0.266
	>6.10	16 (42.1)	34 (55.7)	
Hemoglobin	≤135.0	21 (55.3)	31 (50.8)	0.823
	>135.0	17 (44.7)	30 (49.2)	
Red blood cell	≤4.46	19 (50.0)	32 (52.5)	0.975
	>4.46	19 (50.0)	29 (47.5)	
Platelet	≤215.0	19 (50.0)	30 (49.2)	1.000
	>215.0	19 (50.0)	31 (50.8)	
Neutrophil	≤3.78	22 (57.9)	29 (47.5)	0.426
	>3.78	16 (42.1)	32 (52.5)	
Lymphocyte	≤1.72	23 (60.5)	35 (57.4)	0.921
	>1.72	15 (39.5)	26 (42.6)	
Eosinophil	≤0.12	20 (52.6)	33 (54.1)	1.000
	>0.12	18 (47.4)	28 (45.9)	
Basophil	≤0.03	25 (65.8)	45 (73.8)	0.534
	>0.03	13 (34.2)	16 (26.2)	
Monocyte	≤0.32	22 (57.9)	29 (47.5)	0.426
	>0.32	16 (42.1)	32 (52.5)	
Tumor site	Ocular choroid	2 (5.3)	19 (31.1)	0.004
	Mucosa	8 (21.1)	15 (24.6)	
	Acral skin	28 (73.7)	27 (44.3)	
T stage	T1	2 (5.3)	1 (1.6)	0.610
	T2	2 (5.3)	2 (3.3)	
	T3	5 (13.2)	12 (19.7)	
	T4	29 (76.3)	46 (75.4)	
N stage	N0	28 (73.7)	53 (86.9)	0.311
	N1	4 (10.5)	3 (4.9)	
	N2	4 (10.5)	2 (3.3)	
	N3	2 (5.3)	3 (4.9)	
M stage	M0	38 (100.0)	59 (96.7)	0.694
	M1	0 (0.0)	2 (3.3)	
TNM stage	I	3 (7.9)	4 (6.6)	0.063
	II	23 (60.5)	48 (78.7)	
	III	12 (31.6)	7 (11.5)	
	IV	0 (0.0)	2 (3.3)	
TLN	≤1	27 (71.1)	47 (77.0)	0.667
	>1	11 (28.9)	14 (23.0)	
PLN	≤0	28 (73.7)	54 (88.5)	0.103
	>0	10 (26.3)	7 (11.5)	
BRAF	Negative	5 (13.2)	13 (21.3)	0.571
	Positive	1 (2.6)	1 (1.6)	
	Unknown	32 (84.2)	47 (77.0)	
CyclinD1	Negative	0 (0.0)	6 (9.8)	0.110
	Positive	5 (13.2)	10 (16.4)	
	Unknown	33 (86.8)	45 (73.8)	
HMB45	Negative	2 (5.3)	2 (3.3)	0.833
	Positive	35 (92.1)	58 (95.1)	
	Unknown	1 (2.6)	1 (1.6)	
Ki67	Negative	0 (0.0)	1 (1.6)	0.716
	Positive	37 (97.4)	58 (95.1)	
	Unknown	1 (2.6)	2 (3.3)	
MelanA	Negative	0 (0.0)	1 (1.6)	0.653
	Positive	36 (94.7)	58 (95.1)	
	Unknown	2 (5.3)	2 (3.3)	
P16	Negative	1 (2.6)	4 (6.6)	0.370
	Positive	2 (5.3)	7 (11.5)	
	Unknown	35 (92.1)	50 (82.0)	
P53	Negative	4 (10.5)	10 (16.4)	0.511
	Positive	19 (50.0)	33 (54.1)	
	Unknown	15 (39.5)	18 (29.5)	
S100	Negative	2 (5.3)	0 (0.0)	0.079
	Positive	36 (94.7)	58 (95.1)	
	Unknown	0 (0.0)	3 (4.9)	
MITF	Negative	3 (7.9)	5 (8.2)	0.993
	Positive	11 (28.9)	17 (27.9)	
	Unknown	24 (63.2)	39 (63.9)	
Clark level	I	6 (15.8)	9 (14.8)	0.539
	II	4 (10.5)	4 (6.6)	
	III	4 (10.5)	5 (8.2)	
	IV	8 (21.1)	8 (13.1)	
	V	9 (23.7)	13 (21.3)	
	Unknown	7 (18.4)	22 (36.1)	
Blood vessel invasion	No	36 (94.7)	59 (96.7)	1.000
	Yes	2 (5.3)	2 (3.3)	
Neural invasion	No	38 (100.0)	56 (91.8)	0.180
	Yes	0 (0.0)	5 (8.2)	
Chemotherapy	No	33 (86.8)	50 (82.0)	0.719
	Yes	5 (13.2)	11 (18.0)	
Radiotherapy	No	35 (92.1)	56 (91.8)	1.000
	Yes	3 (7.9)	5 (8.2)	
Targeted therapy	No	30 (78.9)	54 (88.5)	0.315
	Yes	8 (21.1)	7 (11.5)	
Immunotherapy	No	24 (63.2)	44 (72.1)	0.476
	Yes	14 (36.8)	17 (27.9)	

^#^BMI, body mass index; ALT, Alanine aminotransferase; AST, Aspartate aminotransferase; TP, Total protein; ALB, Albumin; GLO, Globularproteins; TBIL, Total bilirubin; DBIL, Direct bilirubin; TBA, Total bile acids; BUN, Blood urea nitrogen; CREA, Creatinine; UA, Uric acid; CO2CP, Carbon dioxide combining power; GLU, Glucose; FDP, Fibrinogen degradation products; INR, international normalized ratio; Fib, Fibrinogen; ESR, Erythrocyte sedimentation rate; CRP, C-reactive protein; TLN, Total lymph node; PLN, Positive lymph node.

### The univariate analysis and multivariate analysis for potential prognostic factors in melanoma patients

Based on the univariate analysis, MC1RN (MC1R expressed in normal tissues adjacent to melanoma tissues), MC1RT (MC1R expressed in melanoma tumor tissue), sex, ESR, tumor site, targeted therapy, and immunotherapy were associated with DFS. Based on the multivariate analysis, MC1RN, MC1RT, sex, ESR, tumor site, targeted therapy, and immunotherapy were the potential prognostic factors for the DFS for melanoma (**Table 2**). Using univariate analysis, MC1RN, MC1RT, sex, tumor site, TLN, PLN, and immunotherapy were associated with OS. Based on multivariate analysis, MC1RN, MC1RT, sex, tumor site, TLN, PLN, and immunotherapy were the potential prognostic factors for OS for melanoma (**Table 3**).

**Table 2:** Univariate and multivariate COX regression model analyses for the prediction of DFS in melanoma patients.

Parameters		P	HR	95% CI		P	HR	95% CI	
				Low	High			Low	High
MC1RN	Low	0.014	1 (Ref.)			0.005	1 (Ref.)		
	High		3.380	1.282	8.910		5.552	1.684	18.303
MC1RT	Low	0.031	1 (Ref.)			0.036	1 (Ref.)		
	High		3.932	1.135	13.624		3.781	1.094	13.064
Sex	Male	0.000	1 (Ref.)			0.002	1 (Ref.)		
	Female		0.075	0.020	0.279		0.290	0.130	0.646
Age	≤58	0.239	1 (Ref.)						
	>58		0.413	0.095	1.802				
Weight	≤60	0.322	1 (Ref.)						
	>60		1.586	0.637	3.951				
Height	≤1.60	0.798	1 (Ref.)						
	>1.60		1.163	0.366	3.693				
BMI	≤23.44	0.811	1 (Ref.)						
	>23.44		1.117	0.449	2.778				
Family history	Yes	0.512	1 (Ref.)						
	No		0.456	0.044	4.758				
ALB	≤39.80	0.881	1 (Ref.)						
	>39.80		0.927	0.342	2.512				
Fib	≤2.88	0.858	1 (Ref.)						
	>2.88		1.089	0.430	2.760				
D Dimer	≤0.38	0.744	1 (Ref.)						
	>0.38		1.231	0.353	4.297				
ESR	≤8.00	0.032	1 (Ref.)			0.007	1 (Ref.)		
	>8.00		4.961	1.145	21.495		5.547	1.600	19.228
CRP	≤3.59	0.367	1 (Ref.)						
	>3.59		0.616	0.215	1.765				
Neutrophil	≤3.78	0.056	1 (Ref.)						
	>3.78		3.582	0.967	13.266				
Lymphocyte	≤1.72	0.191	1 (Ref.)						
	>1.72		2.086	0.693	6.282				
Monocyte	≤0.32	0.156	1 (Ref.)						
	>0.32		0.400	0.113	1.418				
Tumor site	Ocular choroid	0.000	1 (Ref.)			0.000	1 (Ref.)		
	Mucosa	0.000	14.475	3.848	54.449	0.000	21.377	5.326	85.811
	Acral skin	0.064	10.506	0.870	126.841	0.021	5.358	1.288	22.283
TNM stage	I	0.358	1 (Ref.)						
	II	0.462	1.356	0.134	14.324				
	III	0.744	1.553	0.016	19.465				
	IV	0.308	3.506	0.105	62.604				
TLN	≤1	0.088	1 (Ref.)						
	>1		4.901	0.787	30.510				
PLN	≤0	0.073	1 (Ref.)						
	>0		2.623	0.913	7.539				
Blood vessel invasion	No	0.515	1 (Ref.)						
	Yes		0.393	0.024	6.508				
Neural invasion	No	0.925	1 (Ref.)						
	Yes		1.101	0.146	8.293				
Chemotherapy	No	0.923	1 (Ref.)						
	Yes		1.075	0.246	4.700				
Radiotherapy	No	0.461	1 (Ref.)						
	Yes		1.957	0.328	11.670				
Targeted therapy	No	0.022	1 (Ref.)			0.009	1 (Ref.)		
	Yes		5.374	1.278	22.593		2.964	1.317	6.668
Immunotherapy	No	0.001	1 (Ref.)			0.000	1 (Ref.)		
	Yes		6.952	2.198	21.989		8.263	3.655	18.679

^#^MC1RN, MC1R expressed in normal tissues adjacent to melanoma tissues; MC1RT, MC1R expressed in melanoma tumor tissue; BMI, body mass index; ALB, Albumin; Fib, Fibrinogen; ESR, Erythrocyte sedimentation rate; CRP, C-reactive protein; TLN, Total lymph node; PLN, Positive lymph node.

**Table 3:** Univariate and multivariate COX regression model analyses for the prediction of OS in melanoma patients.

Parameters		P	HR	95% CI		P	HR	95% CI	
				Low	High			Low	High
MC1RN	Low	0.024	1 (Ref.)			0.040	1 (Ref.)		
	High		3.051	1.158	8.042		2.243	1.036	4.855
MC1RT	Low	0.012	1 (Ref.)			0.038	1 (Ref.)		
	High		4.737	1.402	16.005		4.309	1.083	17.145
Sex	Male	0.009	1 (Ref.)			0.026	1 (Ref.)		
	Female		0.137	0.031	0.606		0.421	0.197	0.902
Age	≤58	0.746	1 (Ref.)						
	>58		1.334	0.233	7.626				
Weight	≤60	0.425	1 (Ref.)						
	>60		1.451	0.581	3.620				
Height	≤1.60	0.155	1 (Ref.)						
	>1.60		2.372	0.721	7.809				
BMI	≤23.44	0.726	1 (Ref.)						
	>23.44		1.176	0.474	2.915				
Family history	Yes	0.512	1 (Ref.)						
	No		0.456	0.044	4.758				
ALB	≤39.80	0.287	1 (Ref.)						
	>39.80		1.754	0.624	4.932				
Fib	≤2.88	0.997	1 (Ref.)						
	>2.88		0.998	0.395	2.524				
D Dimer	≤0.38	0.378	1 (Ref.)						
	>0.38		0.512	0.115	2.270				
ESR	≤8.00	0.473	1 (Ref.)						
	>8.00		1.737	0.384	7.850				
CRP	≤3.59	0.928	1 (Ref.)						
	>3.59		0.959	0.389	2.365				
Neutrophil	≤3.78	0.915	1 (Ref.)						
	>3.78		1.083	0.252	4.655				
Lymphocyte	≤1.72	0.375	1 (Ref.)						
	>1.72		1.761	0.504	6.148				
Monocyte	≤0.32	0.639	1 (Ref.)						
	>0.32		0.706	0.164	3.031				
Tumor site	Ocular choroid	0.000	1 (Ref.)			0.001	1 (Ref.)		
	Mucosa	0.005	4.999	1.423	17.565	0.007	5.666	1.620	19.815
	Acral skin	0.259	0.256	0.024	2.733	0.38	1.786	0.489	6.521
TNM stage	I	0.322	1 (Ref.)						
	II	0.735	2.016	0.035	17.390				
	III	0.672	2.375	0.043	30.821				
	IV	0.188	3.387	0.214	60.484				
TLN	≤1	0.000	1 (Ref.)			0.000	1 (Ref.)		
	>1		3.541	1.704	7.357		4.639	2.019	10.659
PLN	≤0	0.040	1 (Ref.)			0.004	1 (Ref.)		
	>0		3.121	1.054	9.243		10.173	2.086	49.612
Blood vessel invasion	No	0.980	1 (Ref.)						
	Yes		1.04	0.048	22.361				
Neural invasion	No	0.962	1 (Ref.)						
	Yes		1.05	0.140	7.905				
Chemotherapy	No	0.401	1 (Ref.)						
	Yes		0.429	0.060	3.091				
Radiotherapy	No	0.358	1 (Ref.)						
	Yes		2.496	0.355	17.542				
Targeted therapy	No	0.968	1 (Ref.)						
	Yes		1.033	0.21	5.077				
Immunotherapy	No	0.000	1 (Ref.)			0.000	1 (Ref.)		
	Yes		11.765	3.037	45.587		5.053	2.532	10.082

^#^MC1RN, MC1R expressed in normal tissues adjacent to melanoma tissues; MC1RT, MC1R expressed in melanoma tumor tissue; BMI, body mass index; ALB, Albumin; Fib, Fibrinogen; ESR, Erythrocyte sedimentation rate; CRP, C-reactive protein; TLN, Total lymph node; PLN, Positive lymph node.

### Nomogram construction and validation

**Figure 3:**
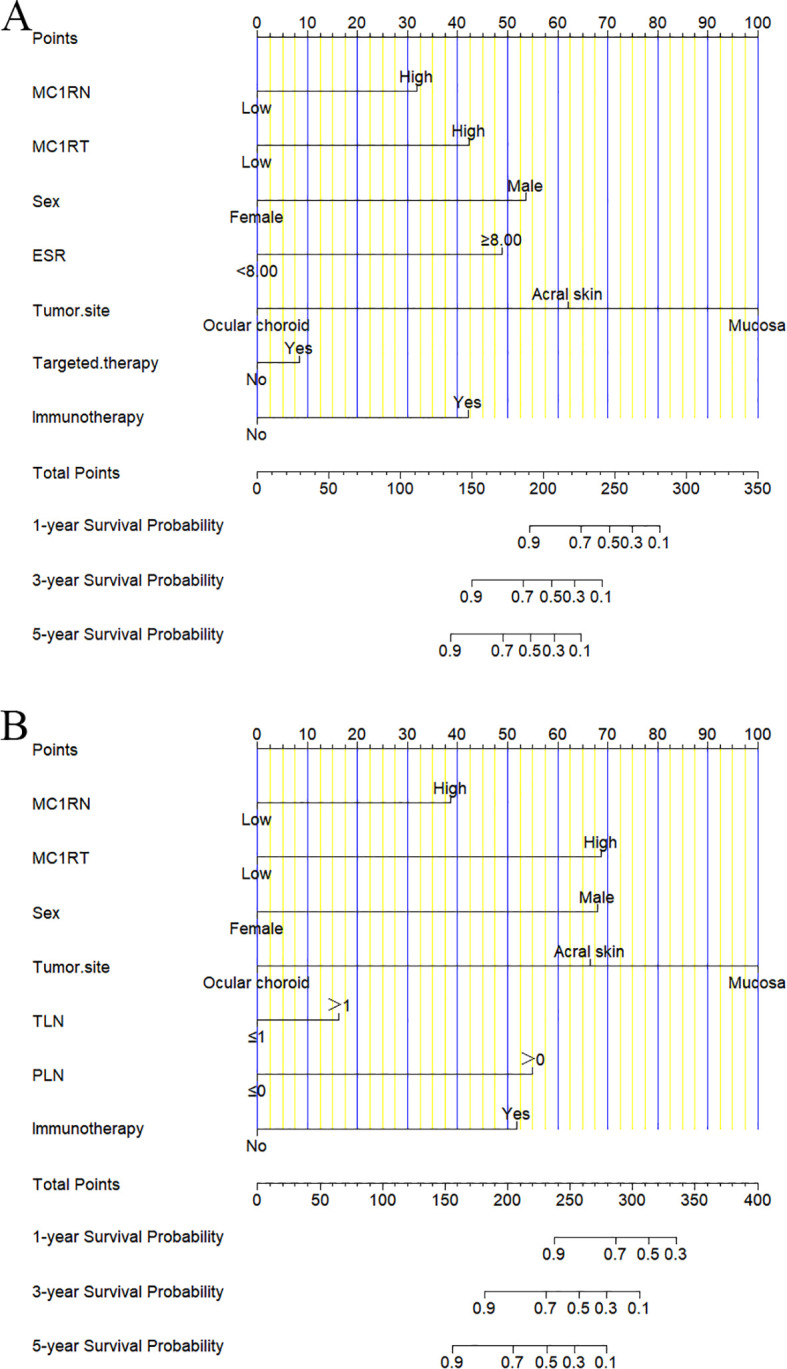
Nomograms for prognosis prediction in melanoma patients. **(A)** One-year, three-year, and five-year nomograms for prognosis prediction for DFS. **(B)** One-year, three-year, and five-year nomograms for prognosis prediction for OS.

For the nomogram predicting DFS, the corresponding scores were calculated based on seven factors, including MC1RN, MC1RT, sex, ESR, tumor site, targeted therapy, and immunotherapy. The scores were further summarized and projected onto the total subscale to predict the probability of the DFS for individual melanoma patients (**Figure 3A**). For the nomogram predicting OS, the corresponding scores were calculated based on seven factors, including MC1RN, MC1RT, sex, tumor site, TLN, PLN, and immunotherapy. These scores were further summarized and projected onto the total subscale to predict the probability of OS for individual melanoma patients (**Figure 3B**).

**Figure 4:**
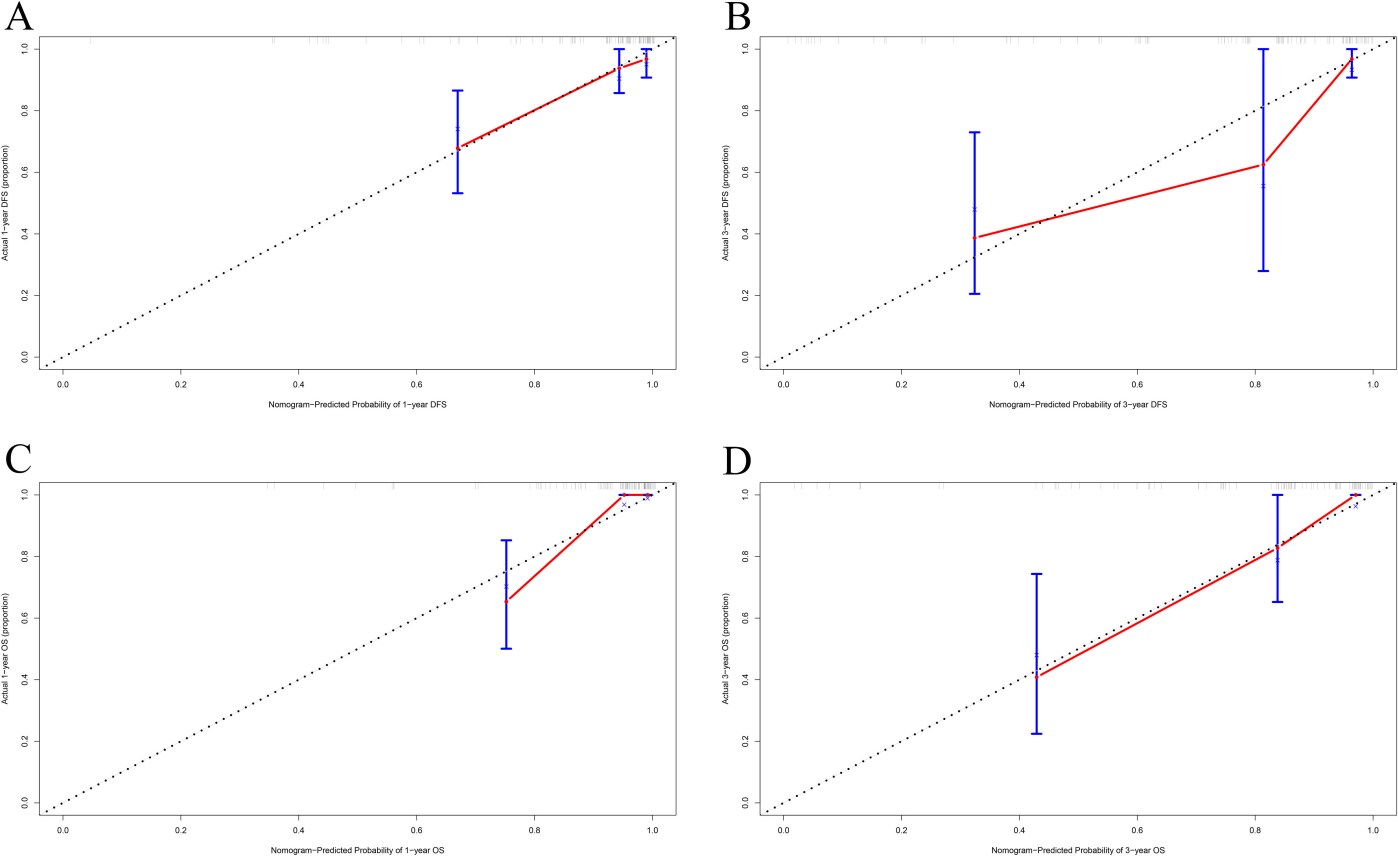
Calibration curves for nomograms in melanoma patients. **(A, B)** One-year and three-year calibration curves for nomograms for prognosis prediction for DFS. **(C, D)** One-year and three-year calibration curves for nomograms for prognosis prediction for OS.

**Figure 5:**
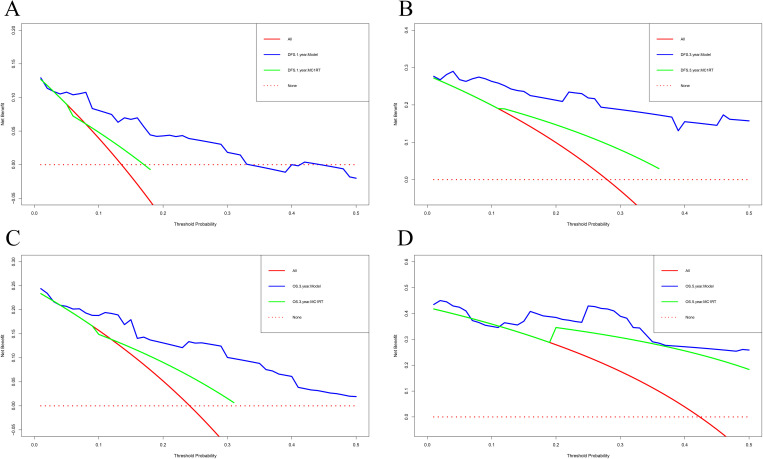
Decision curve analysis for nomograms in melanoma patients using nomogram models and MC1RT. **(A, B)** One-year and three-year decision curve analysis for nomograms for prognosis prediction for DFS. **(C, D)** Three-year and five-year decision curve analysis for nomograms for prognosis prediction for OS.

The calibration curve was used to verify the nomograms, and the results revealed that significant correlation between predictions and actual observations at one year and three years following surgery (**Figures 4A–D**). The decision curve analysis was used to assess the clinical application value of the nomogram model and indicated that the nomogram model predicted DFS and OS in the clinical application better than MC1RT (**Figures 5A–D**).

### Time-dependent ROC drawing and validation

**Figure 6:**
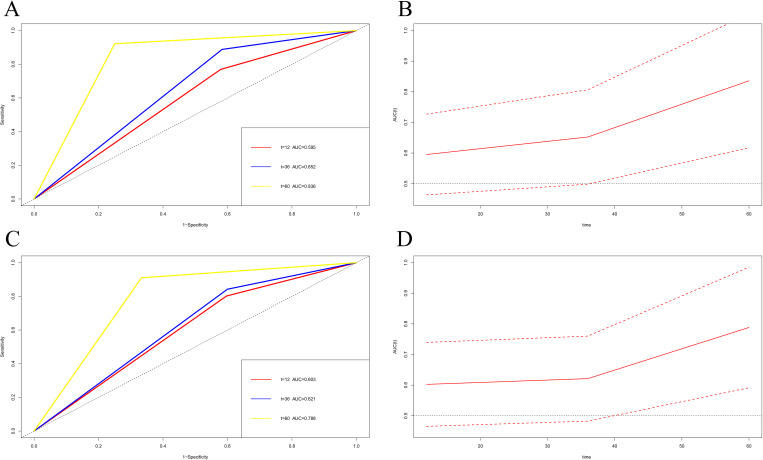
Time-dependent receiver operating characteristic (TDROC) used to analyze the area plots under the receiver operating characteristic curves (AUROCs) for MC1R expression in melanoma tumor tissues from melanoma patients after surgery and follow-up. **(A, C)** Time-dependent AUROCs for DFS and OS. **(B, D)** 95%CI changes of AUROCs for DFS and OS.

Time-dependent ROC curve analysis was used to predict the survival of melanoma patients at different times after surgery using MC1RT. TDROC showed that the AUROC for DFS one year after surgery was 0.595 (95% CI: 0.463-0.727). The AUROC for DFS three years after surgery was 0.652 (95% CI: 0.498-0.807). The AUROC for DFS five years after surgery was 0.836 (95% CI: 0.617-1.000) (**Figures 6A, B**). Furthermore, the TDROC indicated that the AUROC for OS was 0.603 (95% CI: 0.465-0.740) one year after surgery, and the AUROC for OS after surgery at three years was 0.621 (95% CI: 0.482-0.760). Finally, the AUROC for OS after surgery at five years was 0.788 (95% CI: 0.591-0.985) (**Figures 6C, D**).

### Subgroup analysis

We investigated MC1R expression in melanoma tumor tissues from the 63 male melanoma patients. There were 24 cases in the low MC1R expression group and 39 cases in the high MC1R expression group (DFS, χ^2^ = 2.3600, P=0.1245; OS, χ^2^ = 1.9730, P=0.1601) (**Supplementary Figure S3**). Furthermore, we investigated MC1R expression in melanoma tumor tissues from the 36 female melanoma patients. There were 14 cases in the low MC1R expression group and 22 cases in the high MC1R expression group (DFS, χ^2^ = 2.6460, P=0.1038; OS, χ^2^ = 2.1080, P=0.1465) (**Supplementary Figure S4**).

We investigated MC1R expression in melanoma tumor tissues from the 31 melanoma patients who received immunotherapy. There were 14 cases in the low MC1R expression group and 17 cases in the high MC1R expression group (DFS, χ^2^ = 0.4000, P=0.5271; OS, χ^2^ = 0.5556, P=0.4560) (**Supplementary Figure S5**).

Moreover, the expression of MC1R in the melanoma tumor tissues from the acral skin group included 28 cases with low MC1R expression and 27 cases with high MC1R expression (DFS, χ^2^ = 6.158, P=0.0131; OS, χ^2^ = 5.490, P=0.0191) (**Supplementary Figure S6**).

## Discussion

Melanoma is a highly malignant tumor derived from melanocytes, commonly found in the skin, as well as in mucous membranes and internal organs; it occurs more commonly in adults and is rarely observed in children (15). Melanoma can evolve from congenital or acquired benign melanocytic nevi or malignant transformation of developmental nevi (16). Compared with other solid malignant tumors, the age of death in cases of melanoma is lower and the prognosis is poor (17).

Melanocortin receptors including five types of melanocortin receptors (MC1R-MC5R) have been discovered (18). MC1R is playing a major role in skin tone and hair color formation (19). Furthermore, MC1R is related to the proliferation, invasion, migration, and metastasis of melanoma. One study demonstrated that proopiomelanocortin (POMC) could produce α-melanotropin (α-MSH), which exerts its biological functions through MC1R. α-MSH also is closely related to the promotion of tumor development, suggesting that MC1R is a promising target for tumor immunotherapy (20).

The current study analyzed the baseline characteristics of 99 melanoma patients, and utilized immunohistochemistry to detect MC1R expression and its relationship with the prognosis of these melanoma patients. We found that the expression level of MC1R in melanoma tumor tissue was higher than in adjacent normal tissue. According to the MC1R expression by H-Score, patients with high expression of MC1R in melanoma tumor tissue or adjacent normal tissue had survived shorter and worse prognosis. Kansal’s study reported that reducing MC1R expression could inhibit melanoma growth (21). In Su DG’s study, they demonstrated that higher MC1R expression was seen in deeper primary lesions and ulcerated lesions and was related to shorter survival in primary and metastatic tumors (22). This is generally consistent with our findings. Another study also indicated that MC1R was expressed more highly in colon tumor tissues than in adjacent tissues; MC1R was related to colon cancer prognosis, and higher expression of MC1R tended to predict a worse prognosis (23). The expression of MC1R was correlated with several factors, including family history, A/G, DBIL, and tumor site (P<0.05). In early-onset basal cell carcinoma (BCC), a family history of skin cancer was associated with an increased risk of early-onset BCC; and family history remained a strong risk factor for early-onset BCC after adjustment for MC1R genotype (24). Zhang X et al. found that the combination of N-methylation and albumin binding enhanced the labeling of MC1R-targeted radioligands for tumor uptake, which was able to overcome the adverse biological characteristics and dose limitations associated with existing single-molecule therapies, improving treatment efficacy, and demonstrating significant survival advantages in melanoma models (25).

Based on COX regression analysis, we found that MC1RN, MC1RT, sex, ESR, tumor site, targeted therapy, and immunotherapy were the potential prognostic factors for the DFS of melanoma and MC1RN, MC1RT, sex, tumor site, TLN, PLN, and immunotherapy were the potential prognostic factors for the OS of melanoma. Nomograms were assessed to evaluate and predict the survival probability of individualized melanoma patients with different DFS and OS. The nomogram models were validated using calibration curves and demonstrated a high degree of accuracy and consistency between the predicted and actual probabilities at one year and three years following surgery. The nomogram models performed better than MC1RT or MC1RN alone by decision curve analysis. We also applied time-dependent ROC curves to analyze the predictive effects of MC1RT and MC1RN on survival at different times after surgery. With the extension of survival time at any time, the area of the postoperative AUC and 95% CI gradually increased, resulting in more accurate predictions. This preliminary result indicated that the nomogram models exhibited clinical significance.

Research has shown that sex has an impact on melanoma progression, and female patients typically experience significantly better survival than male patients and younger patients perform better than older individuals (26). Our study determined that male melanoma patients experienced significantly shorter postoperative survival times than female melanoma patients. Wendt J’s study demonstrated that MC1R variants contribute differently to melanoma risk in males and females, and was helpful to better classify melanoma risk factors among different sexes (13).

We also found that MC1R was related to the prognosis of melanoma patients receiving immunotherapy. However, due to the small number of melanoma patients receiving immunotherapy, there was no significant correlation between the expression of MC1R in tumor tissues and patient prognosis. A published study of 115 metastatic melanoma patients indicated that the median OS for melanoma patients receiving immunotherapy was 19.0 months, the median OS for melanoma patients receiving conventional chemotherapy was 8.0 months, and that of melanoma patients not receiving treatment was 6.0 months; the intergroup comparison revealed statistical significance (27). Another study suggested that elevated MC1R expression in human melanoma cases was associated with reduced expression of CXCL9/10/11, impaired T cell infiltration, and a poor prognosis based on the GNAS-PKA signaling pathway (28). Zhang Y’s study demonstrated that the bispecific antibody combined MC1R with PD-L1 expressed on melanoma cells could enhance specific antitumor efficacy in a syngeneic B16-SIY melanoma mouse model, and increase infiltrated T cells in the tumor microenvironment (29). Ascierto et al. found that compared with a placebo group, vemurafenib provided a longer DFS survival time in patients with CD8+T cells<1% (HR: 0.56, 95% CI: 0.34-0.92) and a shorter DFS survival time in patients with CD8+T cells ≥1% (HR: 0.77, 95% CI: 0.48-1.22) (30). Furthermore, vemurafenib treatment was observed to provide a longer DFS survival time in patients with PD-L1+ immune cells <5% in tumors (HR: 0.36; 95% CI: 0.24-0.56) and a shorter DFS survival time in patients with PD-L1+ immune cells ≥5% (HR: 0.99, 95% CI: 0.58-1.69) (30). Suzuki T’s study demonstrated that MT-7117 was a novel oral MC1R agonist that induces melanogenesis *in vitro* and *in vivo*, suggesting its potential application for the prevention of phototoxic reactions in patients with photodermatoses (31). Therefore, MC1R might be a potential therapeutic target for tumor immunotherapy.

In the current study, the tumor locations included 21 cases (21.2%) of ocular choroid, 23 cases (23.2%) of mucosa, and 55 cases (55.6%) of acral skin. We conducted an in-depth analysis of the differences in the MC1R expression in melanomas of the ocular choroid, mucosa, and acral skin. Patients with high MC1R expression in melanoma had a shorter survival time in the acral skin group. Research has shown that melanoma location is related to patient prognosis (32). Patients with melanoma occurring on the skin of the limbs have a better prognosis, with a five-year survival rate of 60% for upper limb patients and 57% for lower limb patients. Patients with melanoma occurring on the head are the second most common category, with a five-year survival rate of 53%. Those with melanoma occurring on the trunk have a worse prognosis, with a five-year survival rate of 41%, and the worst five-year survival rate (20-40%) is reported for melanoma arising in the mucosa (32). A study by Tyrrell et al. reviewed the characteristics and prognosis of patients with mucosal melanoma and indicated that patients with mucosal melanoma also exhibited a poor prognosis and short survival time (33). Lian Bin et al. also analyzed the clinical and pathological characteristics of 232 patients with advanced mucosal melanoma; nasal and oral mucosa were the most common sites of melanoma, and M stage and LDH level at the time of treatment were independent prognostic factors for OS (34).

There are several limitations associated with this study. First, it was a single institution retrospective study. Our findings necessitate further prospective validation through multicenter studies involving independent patient groups. Increasing the sample size, particularly for subgroup analyses, would enhance the statistical power and reliability of the findings. Second, the expression of MC1R was only determined using immunohistochemistry, and the underlying molecular biological mechanisms have not been thoroughly investigated. Additionally, we plan to conduct molecular mechanistic investigations using *in vitro* and *in vivo* models to further elucidate the underlying mechanisms of MC1R-related effects observed in the current study, especially in MC1R activity modulate PKA signaling, immune suppression pathways. Finally, the nomogram models that were constructed need additional validation. The more patients should be enrolled to validate. And the more data collection of the enrolled patients, including clinical, pathological, and follow-up data, would improve data quality and reduce bias.

## Conclusions

In summary, we analyzed the relationship between MC1R expression and melanoma, and determined that low MC1R expression in melanoma tumor tissues and adjacent normal tissue might be beneficial for the prognosis of melanoma patients. The MC1R expression was also concerned with melanoma location. An in-depth analysis of potential prognostic factors affecting melanoma, such as MC1R, tumor site, sex, and immunotherapy, also could affect the prognosis of melanoma patients.

## Data Availability

The original contributions presented in the study are included in the article/**Supplementary Material**. Further inquiries can be directed to the corresponding author.

## References

[1] Carvajal RD Sacco JJ Jager MJ Eschelman DJ Olofsson Bagge R Harbour JW. Advances in the clinical management of uveal melanoma. Nat Rev Clin Oncol. (2023) 20:99–115. doi: 10.1038/s41571-022-00714-1 36600005

[2] Arnold M Singh D Laversanne M Vignat J Vaccarella S Meheus F. Global burden of cutaneous melanoma in 2020 and projections to 2040. JAMA Dermatol. (2022) 158:495–503. doi: 10.1001/jamadermatol.2022.0160 35353115 PMC8968696

[3] Guo W Ren G Sun M Kong Y Wang L Bu R. Expert consensus on clinical diagnosis and treatment for Chinese oral mucosal melanoma. China J Oral Maxillofac Surg. (2021) 19:481–8. doi: 10.19438/j.cjoms.2021.06.001

[4] Wu Y Wang Y Wang L Yin P Lin Y Zhou M. Burden of melanoma in China, 1990-2017: Findings from the 2017 global burden of disease study. Int J Cancer. (2020) 147:692–701. doi: 10.1002/ijc.v147.3 31671209

[5] Maheshwari A Finger PT. Laser treatment for choroidal melanoma: Current concepts. Surv Ophthalmol. (2023) 68:211–24. doi: 10.1016/j.survophthal.2022.05.002 35644256

[6] Li Y Li Y Wang B Hu C Chen Y Zhang G. Research progress in biological therapy for melanoma. Chin J Gerontology. (2022) 42:1235–9. doi: 10.3969/j.issn.1005-9202.2022.05.060

[7] Chen S Zhu B Yin C Liu W Han C Chen B. Palmitoylation-dependent activation of MC1R prevents melanomagenesis. Nature. (2017) 549:399–403. doi: 10.1038/nature23887 28869973 PMC5902815

[8] Peng L Chang J Liu X Lu S Ren H Zhou X. MC1R is a prognostic marker and its expression is correlated with MSI in colorectal cancer. Curr Issues Mol Biol. (2021) 43:1529–47. doi: 10.3390/cimb43030108 PMC8929037 34698109

[9] Chelakkot VS Thomas K Romigh T Fong A Li L Ronen S. MC1R signaling through the cAMP-CREB/ATF-1 and ERK-NFκB pathways accelerates G1/S transition promoting breast cancer progression. NPJ Precis Oncol. (2023) 7:85. doi: 10.1038/s41698-023-00437-1 37679505 PMC10485002

[10] Zhou X Chang J Peng L Liu X Yu F Xu J. MC1R is highly expressed in esophageal squamous cell carcinoma. J South Med Univ. (2022) 42:1552–9. doi: 10.12122/j.issn.1673-4254.2022.10.16 PMC9637506 36329591

[11] Ruano APC Gadelha Guimarães AP Braun AC Flores BCTCP Tariki MS Abdallah EA. Fusion cell markers in circulating tumor cells from patients with high-grade ovarian serous carcinoma. Int J Mol Sci. (2022) 23:14687. doi: 10.3390/ijms232314687 36499015 PMC9740150

[12] Chen S Han C Miao X Li X Yin C Zou J. Targeting MC1R depalmitoylation to prevent melanomagenesis in redheads. Nat Commun. (2019) 10:877. doi: 10.1038/s41467-019-08691-3 30787281 PMC6382811

[13] Wendt J Mueller C Rauscher S Fae I Fischer G Okamoto I. Contributions by MC1R variants to melanoma risk in males and females. JAMA Dermatol. (2018) 154:789–95. doi: 10.1001/jamadermatol.2018.1252 PMC6128491 29898205

[14] Yang J Xu J Gonzalez R Lindner T Kratochwil C Miao Y. 68Ga-DOTA-GGNle-CycMSHhex targets the melanocortin-1 receptor for melanoma imaging. Sci Transl Med. (2018) 10:eaau4445. doi: 10.1126/scitranslmed.aau4445 30404861 PMC6383514

[15] Gao X Zhao X Han Z. Research progress on the association of autophagy with invasion and metastasis of Malignant melanoma. Diagnosis Ther J Dermato-Venereology. (2024) 31:118–22. doi: 10.3969/j.issn.1674-8468.2024.02.009

[16] Qiu J Huang J Xu S Zhou Y Huang P Fu L. Progress in the treatment of Malignant melanoma of the skin. Gansu Med J. (2023) 42:969–71. doi: 10.15975/j.cnki.gsyy.2023.11.003

[17] Wu S Wang Y Zheng K Hang H Kang J Yu H. Prognosis and influencing factors of patients with Malignant melanoma. Chin Gen Pract. (2024) 27:942–7. doi: 10.12114/j.issn.1007-9572.2023.0307

[18] Liu S Pei S Wen K Peng A Ma H Huang H. MC1R-targeted NIR-II aggregation-induced emission nanoparticles for melanoma imaging. Sci China (Materials). (2023) 66:4100–8. doi: 10.1007/s40843-023-2570-1

[19] Yang X Luo Z Li J. Effect of MC1R on proliferation and apoptosis of vitiligo melanocytes. China Med And Pharm. (2023) 13:25–9. doi: 10.20116/j.issn2095-0616.2023.23.06

[20] Xu Y Yan J Tao Y Qian X Zhang C Yin L. Pituitary hormone α-MSH promotes tumor-induced myelopoiesis and immunosuppression. Science. (2022) 377:1085–91. doi: 10.1126/science.abj2674 35926007

[21] Kansal RG McCravy MS Basham JH Earl JA McMurray SL Starner CJ. Inhibition of melanocortin 1 receptor slows melanoma growth, reduces tumor heterogeneity and increases survival. Oncotarget. (2016) 7:26331–45. doi: 10.18632/oncotarget.v7i18 PMC5041983 27028866

[22] Su DG Djureinovic D Schoenfeld D Marquez-Nostra B Olino K Jilaveanu L. Melanocortin-1 receptor expression as a marker of progression in melanoma. JCO Precis Oncol. (2024) 8:e2300702. doi: 10.1200/PO.23.00702 38662983 PMC11513442

[23] Zhu S Zou M Li C Tang Y Luo H Dong X. MC1R regulates T regulatory cell differentiation through metabolic reprogramming to promote colon cancer. Int Immunopharmacol. (2024) 138:112546. doi: 10.1016/j.intimp.2024.112546 38917522

[24] Berlin NL Cartmel B Leffell DJ Bale AE Mayne ST Ferrucci LM. Family history of skin cancer is associated with early-onset basal cell carcinoma independent of MC1R genotype. Cancer Epidemiol. (2015) 39:1078–83. doi: 10.1016/j.canep.2015.09.005 PMC4679454 26381319

[25] Zhang X Chen F Turker MZ Ma K Zanzonico P Gallazzi F. Targeted melanoma radiotherapy using ultrasmall 177Lu-labeled α-melanocyte stimulating hormone-functionalized core-shell silica nanoparticles. Biomaterials. (2020) 241:119858. doi: 10.1016/j.biomaterials.2020.119858 32120314 PMC7171978

[26] Kudura K Basler L Nussbaumer L Foerster R. Sex-related differences in metastatic melanoma patients treated with immune checkpoint inhibition. Cancers (Basel). (2022) 14:5145. doi: 10.3390/cancers14205145 36291928 PMC9600302

[27] Matsui Y Sasaki J Takatsuka S Takenouchi T. Trends in the prognosis of metastatic melanoma in the era of targeted therapy and immunotherapy: A single-institution survey in Japan. J Dermatol. (2021) 48:75–9. doi: 10.1111/1346-8138.15642 33063902

[28] Carlino MS Larkin J Long GV. Immune checkpoint inhibitors in melanoma. Lancet. (2021) 398:1002–14. doi: 10.1016/S0140-6736(21)01206-X 34509219

[29] Zhang Y Fang C Wang RE Wang Y Guo H Guo C. A tumor-targeted immune checkpoint blocker. Proc Natl Acad Sci U.S.A. (2019) 116:15889–94. doi: 10.1073/pnas.1905646116 PMC6689898 31332018

[30] Ascierto PA Lewis KD Di Giacomo AM Demidov L Mandalà M Bondarenko I. Prognostic impact of baseline tumour immune infiltrate on disease-free survival in patients with completely resected, BRAFv600 mutation-positive melanoma receiving adjuvant vemurafenib. Ann Oncol. (2020) 31:153–9. doi: 10.1016/j.annonc.2019.10.002 31912791

[31] Suzuki T Kawano Y Matsumoto A Kondo M Funayama K Tanemura S. Melanogenic effect of dersimelagon (MT-7117), a novel oral melanocortin 1 receptor agonist. Skin Health Dis. (2021) 2:e78. doi: 10.1002/ski2.78 35665216 PMC9060023

[32] Wu X Feng Y Zeng C Wang M. Analysis of prognostic factors for survival in patients with head and neck mucosal melanoma. J Clin Otorhinolarynglolgy Head Neck Surg. (2020) 34:647–51. doi: 10.13201/j.issn.2096-7993.2020.07.017 PMC10133107 32791644

[33] Tyrrell H Payne M. Combatting mucosal melanoma: recent advances and future perspectives. Melanoma Manag. (2018) 5:MMT11. doi: 10.2217/mmt-2018-0003 30459941 PMC6240847

[34] Lian B Zheng N Chi Z Zhou L Sheng X Si L. Curative effect of the 1st line and the 2nd line therapies for the 232 patients with advanced mucosal melanoma and factors effecting prognosis of the patients. Chin J Cancer Biother. (2017) 24:253–8. doi: 10.3872/j.issn.1007-385X.2017.03.007

