# Supplementary material for: Melanocortin-1 receptor expression as a predictive factor for postoperative outcomes in melanoma patients: a retrospective study
Source: Front Immunol. 2025 Mar 27;16:1570502. doi: 10.3389/fimmu.2025.1570502 (PMC11983465; doi:10.3389/fimmu.2025.1570502)
Supplement: Supplementary file 1 [file DataSheet1.docx]

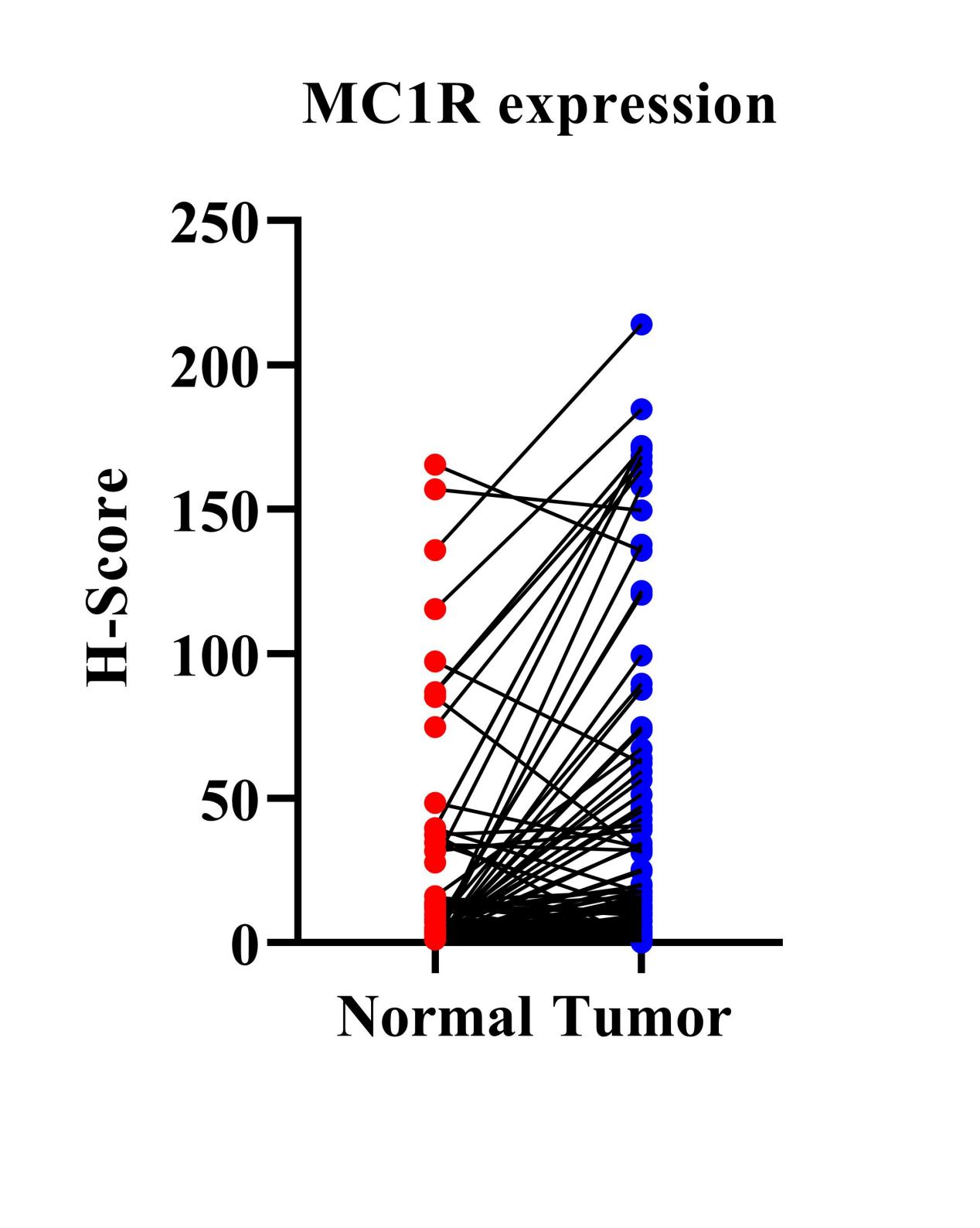


**Figure S1** The expression of MC1R in melanoma tumor tissues and adjacent normal tissues.


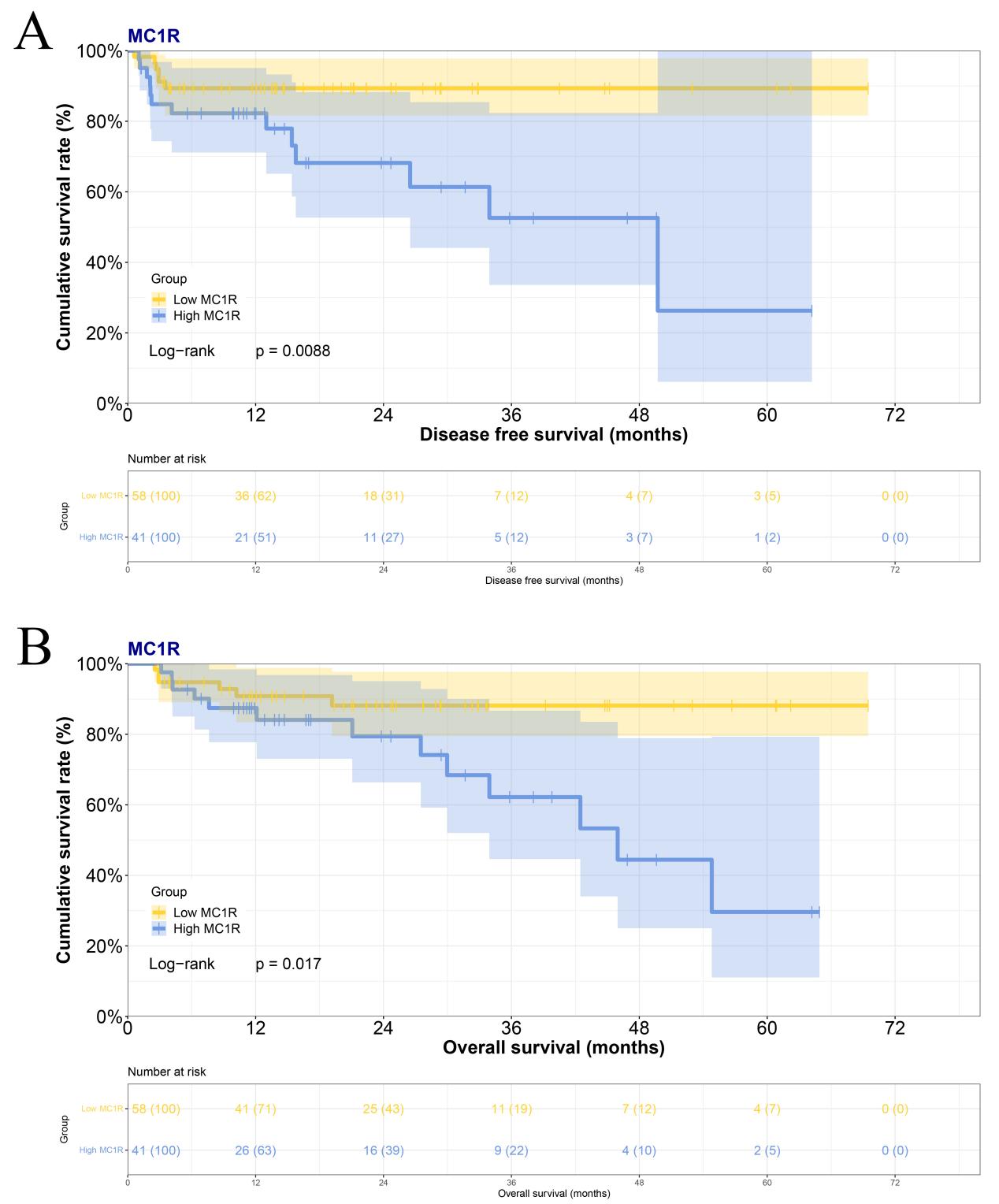


**Figure S2** The association between the expression of MC1R in normal tissues adjacent to melanoma tissues and the prognosis in melanoma patients.

**
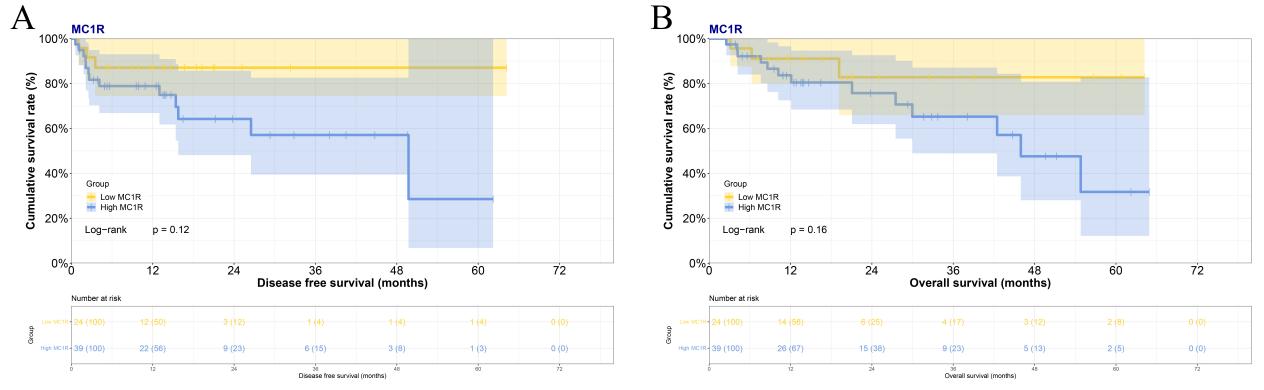
**

**Figure S3** The association between MC1R expression in melanoma tumor tissue and the prognosis in male melanoma patients for A) DFS and B) OS.


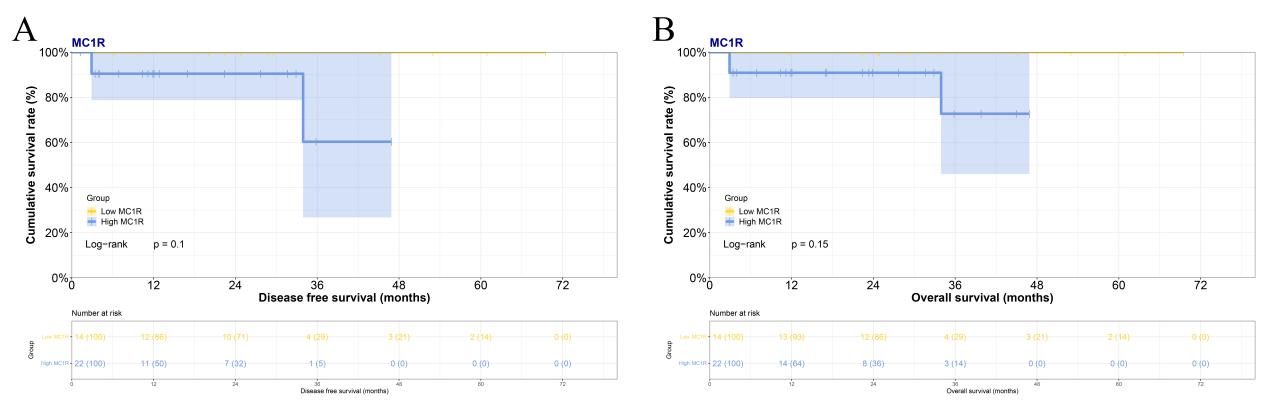


**Figure S4** The association between MC1R expression in melanoma tumor tissue and the prognosis in female melanoma patients for A) DFS and B) OS.


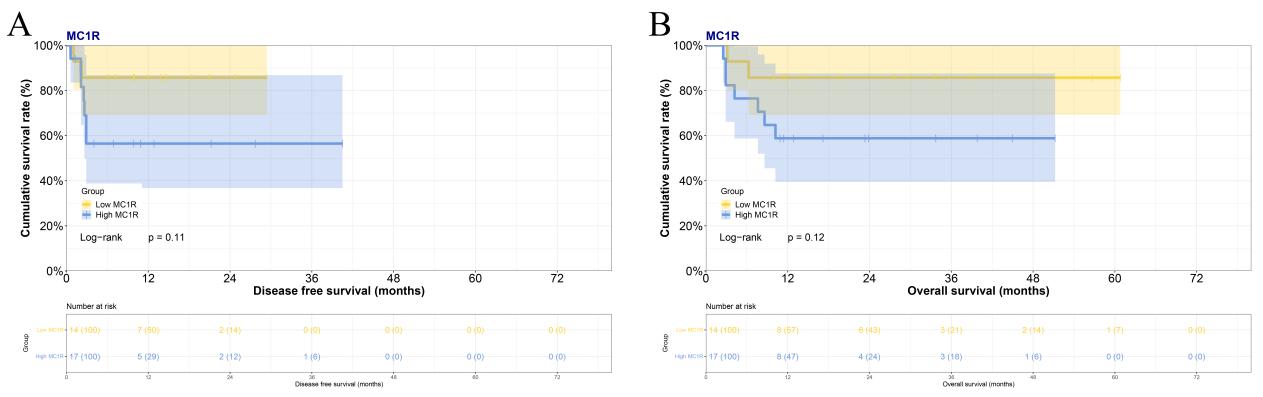


**Figure S5** The association between MC1R expression in melanoma tumor tissue and the prognosis in melanoma patients who received immunotherapy for A) DFS and B) OS.


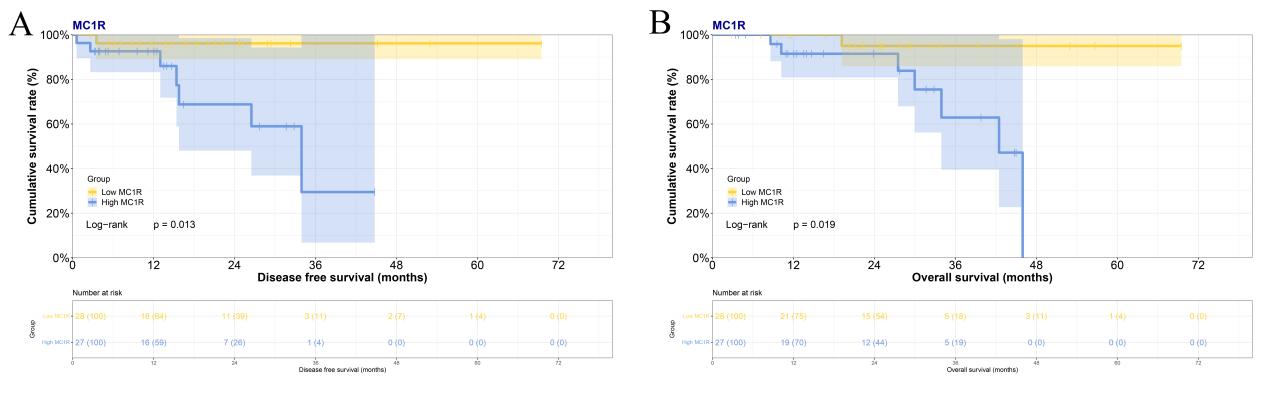


**Figure S6** The association between MC1R expression in melanoma tumor tissue and the prognosis in limb skin melanoma patients for A) DFS and B) OS.
